# Differences between Risk Factors Associated with Tuberculosis Treatment Abandonment and Mortality

**DOI:** 10.1155/2015/546106

**Published:** 2015-10-27

**Authors:** Nathália Mota de Faria Gomes, Meire Cardoso da Mota Bastos, Renata Magliano Marins, Aline Alves Barbosa, Luiz Clóvis Parente Soares, Annelise Maria de Oliveira Wilken de Abreu, João Tadeu Damian Souto Filho

**Affiliations:** ^1^Faculdade de Medicina de Campos (FMC), 28035-581 Campos dos Goytacazes, RJ, Brazil; ^2^Programa de Controle da Tuberculose, Centro de Referência Augusto Guimarães, 28085-500 Campos dos Goytacazes, RJ, Brazil; ^3^Instituto Federal de Educação, Ciência e Tecnologia Fluminense (IFF), 28060-010 Campos dos Goytacazes, RJ, Brazil

## Abstract

*Objectives*. To identify the risk factors that were associated with abandonment of treatment and mortality in tuberculosis (TB) patients. *Methods*. This study was a retrospective longitudinal cohort study involving tuberculosis patients treated between 2002 and 2008 in a TB reference center. *Results*. A total of 1,257 patients were evaluated, with 69.1% men, 54.4% under 40 years of age, 18.9% with extrapulmonary disease, and 9.3% coinfected with HIV. The risk factors that were associated with abandonment of treatment included male gender (OR = 2.05; 95% CI = 1.15–3.65) and nonadherence to previous treatment (OR = 3.14; 95% CI = 1.96–5.96). In addition, the presence of extrapulmonary TB was a protective factor (OR = 0.33, 95% CI = 0.14–0.76). The following risk factors were associated with mortality: age over 40 years (OR = 2.61, 95% CI = 1.76–3.85), coinfection with HIV (OR = 6.01, 95% CI = 3.78–9.56), illiteracy (OR = 1.88, 95% CI = 1.27–2.75), the presence of severe extrapulmonary TB (OR = 2.33, 95% CI = 1.24–4.38), and retreatment after relapse (OR = 1.95, 95% CI = 1.01–3.75). *Conclusions*. Male gender and retreatment after abandonment were independent risk factors for nonadherence to TB treatment. Furthermore, age over 40 years, coinfection with HIV, illiteracy, severe extrapulmonary TB, and retreatment after relapse were associated with higher TB mortality. Therefore, we suggest the implementation of direct measures that will control the identified risk factors to reduce the rates of treatment failure and TB-associated mortality.

## 1. Introduction

Tuberculosis (TB) still represents a major public health problem, especially in developing countries. TB is an infectious disease that is difficult to control because the disease is transmitted through the air. The etiological agent of TB is* Mycobacterium tuberculosis* (MTB), and the disease is characterized by granulomas and the presence of a cell-mediated hypersensitivity reaction [1].

TB mainly affects economically active age groups, which causes problems for the individual as well as community health and results in higher public spending [2]. An estimated one-third of the world's population is infected with TB. In 2011, TB affected 8.4 million people, and the disease caused 1.4 million deaths worldwide [3].

Currently, Brazil ranks fourteenth among the 22 high-burden countries for TB. In 2011, the TB incidence in Brazil was 36 cases per 100,000 inhabitants, and 69,245 new cases were recorded. TB is a major cause of morbidity and mortality in Brazil. This disease is the fourth leading cause of death among infectious diseases, with the estimated annual death rate of 2.4 cases per 100,000 inhabitants in 2010 [3].

The main risk factors that have been identified as predictors of increased mortality in individuals with TB are as follows: irregular or inadequate treatment, delayed diagnosis, multidrug resistance, coinfection with human immunodeficiency virus (HIV), and advanced age [4]. Nonadherence to treatment is a major problem in the management of TB and in the treatment of any chronic disease. TB has characteristics that affect treatment adherence and creates a health challenge. In addition, the treatment of TB is long-term one and requires the administration of various medications. Moreover, despite the frequent adverse effects of the treatment, patients feel physically well before the end of the medication cycle and are thus tempted to abandon treatment [5].

Low adherence is the major cause of treatment failure and drug resistance. Previous studies have demonstrated that the expense of traveling to treatment centers, the male gender, patients with little information about the disease, difficult communication with patients, alcoholism, and homelessness are the main determinants of nonadherence to TB treatment [6]. Patient adherence to standard treatment in developing countries is estimated to be less than 40% [7].

The aim of this study was to determine the epidemiologic profile of TB patients who were undergoing treatment in the city of Campos dos Goytacazes, Rio de Janeiro (RJ), by analyzing the risk factors that were associated with abandonment of treatment and mortality.

## 2. Materials and Methods

A retrospective longitudinal cohort study was conducted by the Tuberculosis Control Program (TCP) at the Augusto Guimarães Reference Center, Municipal Health Department, in Campos dos Goytacazes, RJ. This city is located in the northern region of RJ and is to be the second largest rural city in the state and the tenth largest rural city in Brazil, with a population of 468,087 inhabitants in 2011 according to the Brazilian Institute of Geography and Statistics (Instituto Brasileiro de Geografia e Estatística, IBGE). However, the population is now estimated to exceed 600,000 inhabitants due to the development of the oil industry.

A total of 1,257 medical records from patients, including both genders and all ages, who were treated at the TCP from 01/01/2002 to 12/31/2008 were evaluated. A protocol that was adapted from the Notifiable Diseases Information System (Sistema de Informações de Agravos de Notificação, SINAN) [8], which is a database of the Ministry of Health of Brazil, was used for data collection. This protocol included the collection of information on sociodemographic, epidemiologic, clinical, and laboratory data. The following variables were studied: age (over or under 40 years); gender (male and female); level of education (literate and illiterate); address (urban or rural); TB involvement upon entry into treatment or closure of the case [9]; institutionalization (detention facilities and nursing homes); clinical type (pulmonary and extrapulmonary); comorbidities (alcoholism, smoking, cardiovascular disease, diabetes mellitus (DM), neoplasia, lung diseases, and coinfection with HIV); chest radiography (suspicious, normal, or not performed); tuberculin skin test (TST); sputum smear microscopy; sputum culture; and histopathology.

The definition of the TB cases that were treated by the TCP was based on the following World Health Organization (WHO) classification: new case, treatment after abandonment, treatment after relapse, and transferred-in cost.

Extrapulmonary TB cases were defined as having compatible histopathology or positive culture for MTB associated with a negative sputum smear and culture in respiratory secretions [10, 11]. In this study, the following types of TB were considered to be severe extrapulmonary TB: meningeal, miliary, pericardial, peritoneal, bilateral or extensive pleural effusion, spinal, genitourinary, and intestinal types [9].

Statistical analyses were performed to evaluate the association between the characteristics of the TB patients and the risk for treatment failure, which was based on two outcomes: abandonment of treatment and mortality. In the univariate analysis, the chi-squared test or Fisher's exact test was used for the categorical variables. The variables with *p* < 0.10 in the univariate analysis were included in a multivariate logistic regression model. A *p* < 0.05 was considered statistically significant. The data were analyzed using SPSS 22.0 for Windows (Statistical Package for the Social Sciences, Chicago, IL, USA).

The study was reviewed and approved by the Research Ethics Committee of Faculdade de Medicina de Campos, Campos dos Goytacazes, Rio de Janeiro, Brazil. In this study, institutional consent was obtained for record review and all patient information was anonymized and deidentified prior to analysis.

## 3. Results

A total of 1,257 medical records were evaluated from subjects who were treated by the TCP from 2002 to 2008. The clinical and epidemiologic characteristics of the patients are shown in Table 1. The sample consisted of 868 (69.1%) men and 389 (30.9%) women, and the gender ratio was 2.2 : 1, male to female. In total, 673 (54.4%) patients were under 40 years of age, 314 (25%) patients were illiterate, and 1,107 (88.1%) patients lived in an urban area.

Regarding the TB type, 1,020 (81.1%) patients had pulmonary disease and 237 (18.9%) patients extrapulmonary disease. Among those with extrapulmonary disease, pleural TB and lymph node were the most prevalent types and represented 8.2% and 4.9% of the cases, respectively. The presence of coinfection with HIV was observed in 117 (9.3%) individuals. Overall, 842 (67%) individuals had HIV serological results available, 30 (2.4%) individuals had HIV serology that was still being processed, and 386 (30.7%) individuals were not screened for HIV. Among the patients with extrapulmonary TB, 33 (28.2%) had serological results that were positive for HIV. The univariate analysis revealed a significant association between extrapulmonary TB and HIV infection (*p* = 0.006; OR = 1.81, 95% CI 1.15–2.84).

Table 2 shows the analysis of the risk factors that were associated with abandonment of treatment. In the univariate analysis, male gender, alcoholism and a history of previous abandonment of treatment correlated with an increased risk of non-adherence to current treatment, whereas the presence of extrapulmonary TB, diabetes and other comorbidities were found to be protective factors. However, in the multivariate analysis, the variables that remained significant risk factors for abandonment of treatment were male gender (OR = 2.05; 95% CI 1.15–3.65) and non-adherence to previous treatment (OR = 3.14; 95% CI 1.96–5.96). Having extrapulmonary TB was found to be a protective factor (OR = 0.33; 95% CI 0.14–0.76).

The risk factors that were associated with mortality during TB treatment are presented in Table 3. In the univariate and multivariate analyses, the risk factors that were associated with a higher mortality were as follows: age over 40 years (OR = 2.61; 95% CI 1.76–3.85), coinfection with HIV (OR = 6.01; 95% CI 3.78–9.56), illiteracy (OR = 1.88; 95% CI 1.27–2.75), the presence of severe extrapulmonary TB (OR = 2.33; 95% CI 1.24–4.38), and retreatment after relapse (OR = 1.95; 95% CI 1.01–3.75). Comorbidities and institutionalization were correlated with mortality in the univariate analysis but lost statistical significance in the multivariate analysis.

## 4. Discussion

In most countries, greater numbers of men are diagnosed with TB than women, and men have a higher death rate from TB. This quantitative difference between the sexes is partly due to epidemiological factors, especially the risk of exposure to the infection and the progression from infection to the disease stage [12].

In this study, the statistically significant risk factors that were associated with treatment failure included male gender and retreatment after treatment abandonment, whereas extrapulmonary TB was a protective factor against treatment failure.

The high prevalence of TB in males and the poor adherence to treatment in males may be due to increased exposure to situations of risk for MTB infection, such as interpersonal and social interaction for socioeconomic and cultural reasons. In addition, men may delay seeking treatment at health clinics when their physical conditions worsen. By contrast, women demonstrated a greater adherence to TB treatment, and female gender was not associated with the risk factors for death [13, 14].

Resistance that arises from the irregular intake of medication depends on several cycles of bacterial death (when the drugs are ingested) and bacterial growth (when they are suspended). In each cycle, there is a selection that favors drug-resistant mutants, which is detrimental to sensitive populations of patients [15]. Initially, resistance occurs to a medication in the treatment regimen, followed by the development of resistance to other medications, leading to multiresistance [16]. This scenario of bacterial resistance combined with the possible social vulnerability of the TB carrier leads to treatment failure in the outcomes of these individuals.

Extrapulmonary TB is a protective factor against treatment failure because this TB type is associated with less drug resistance. Studies that were conducted in Europe demonstrated favorable outcomes in 81% and 68% of patients with extrapulmonary TB [17, 18]. The results from these studies corroborate the results that were found in this study. In human TB disease, cavitary lung lesions contain mycobacteria populations that range from 107 to 108 bacilli, whereas the population ranges from 102 to 104 bacilli in hardened caseous lesions [19]. Drug resistance is more common in the cavitary type of TB [19, 20]. An increase in the bacterial population results in an increased probability of resistance in pretreatment. In most types of extrapulmonary TB, the initial bacilli population is smaller than that in cavitary pulmonary TB; therefore, the probability that preexisting resistant mutants are present is lower.

Previous studies have found that alcohol abuse [21] and comorbidities, especially DM [22], are the relevant risk factors that are associated with treatment failure. However, we did not find these same associations in our analysis.

Regarding the risk factors that were associated with mortality, we found that the following variables were relevant: age over 40 years, illiteracy, severe extrapulmonary TB, coinfection with HIV, and retreatment after relapse.

Advanced age represents a factor that is associated with an increased risk of death [23]. In this study, advanced age was associated with an aging population and the effectiveness of the TB control programs, which contributed to the reduction of mortality in the younger age group. Furthermore, individuals over 40 years of age have a greater number of associated comorbidities that require the use of other medications, which may predispose these individuals to the irregular intake of specific medications and enable drug resistance. Therefore, directly observed treatment (DOT) should be established for these patients to decrease abandonment of treatment, mortality, and multidrug resistance.

A lack of education is correlated with poor social conditions, lower perception of health problems, less self-care, and delay in seeking health services. Even when patients are on treatment, a lack of education can lead to the abandonment and misuse of medications. Because TB is a social disease, there is a greater incidence of cases and deaths in the social classes with lower socioeconomic status and less education [24].

Retreatment after a relapse may occur for exogenous reinfection or endogenous reactivation of pulmonary or extrapulmonary lesions. A study that was conducted in Southern Brazil [25] found that the recurrence rate correlated with the irregular use of medications. An incomplete bacteriological cure is the most important cause of endogenous reactivation, which is usually caused by the irregular intake of medications. Excluding the presence of preexisting resistance, reactivation may result from the use of regimens with low bactericidal potency, an inadequate length of treatment, an underdose in the drug prescription, or an inappropriate choice of medication [26].

The improper use of medication increases the relapse rate and the probability of drug resistance as demonstrated by previous studies. These effects can lead to a higher rate of mortality in individuals who undergo retreatment compared with treatment-naïve patients and a higher rate of treatment failure in individuals who undergo retreatment after treatment abandonment as indicated in this study.

The Acquired Immune Deficiency Syndrome (AIDS) epidemic has changed TB prognoses because coinfection with HIV has a greater impact on TB mortality than on TB incidence [27]. Studies have indicated that HIV-TB coinfection increases the chance that patients receive inadequate treatment, which leads to multidrug resistance and increased mortality [28, 29].

In our study, we found a strong association between extrapulmonary TB and HIV infection. Extrapulmonary TB is mainly the result of the reactivation of an outbreak of TB after hematogenous or lymphatic dissemination [30]. HIV patients have natural killer cells that are less cytotoxic [31]; therefore, patients with this immunodeficiency are more likely to develop extrapulmonary TB.

We found that severe extrapulmonary TB is an important risk factor that was associated with increased mortality because this disease may be associated with a greater systemic impact. In addition, severe extrapulmonary TB represents a diagnostic challenge for physicians because the diagnoses and treatments are often delayed [32].

The characteristics of the population in this study are quite similar to those presented in studies involving individuals with tuberculosis throughout Brazil. It is mainly represented by young men with low educational level, similar frequencies of institutionalization, extrapulmonary manifestations, and HIV coinfection [33]. This fact allows us to suspect that the risk factors identified in this study can be used in the evaluation of patients with TB in other parts of Brazil and countries with similar socioeconomic characteristics.

Similar to the analysis conducted in this study, an analysis of the risk factors in different countries and regions is important to detect treatment failure and suggest specific changes in the therapeutic approach to TB. Multidisciplinary patient care, emphasis on patient care, health education using DOT, socioeconomic development, and expansion of HIV serological testing will reduce the current rates of treatment abandonment and mortality in TB.

A limitation of this study is the retrospective design of the analysis because only the risk factors that were identified in the medical records were evaluated. However, this lack of data is common in retrospective studies and its impact was minimized by the availability of standardized medical records and the systematic care of the patients, which was centralized.

## 5. Conclusion

This study revealed that male gender and retreatment after treatment abandonment are independent risk factors for nonadherence to TB treatment. In addition, age over 40 years, coinfection with HIV, illiteracy, severe extrapulmonary TB, and retreatment after relapse were significantly associated with increased TB-related mortality. Therefore, we suggest the implementation of direct measures that will control the identified risk factors to reduce the rates of treatment abandonment and death during TB treatment.

## Figures and Tables

**Table 1:** Characteristics of patients with tuberculosis.

Characteristics	Study population	
	*n* = 1257	(%)
Year of diagnosis		
2002	200	(15.9)
2003	195	(15.5)
2004	198	(15.8)
2005	173	(13.8)
2006	179	(14.3)
2007	142	(11.3)
2008	169	(13.5)
Age^*∗*^		
<40 years	673	(54.4)
>40 years	565	(45.6)
Gender		
Female	389	(30.9)
Male	868	(69.1)
HIV coinfection		
No	1140	(90.7)
Yes	117	(9.3)
Alcoholic		
No	1122	(89.3)
Yes	135	(10.7)
Literacy		
Yes	943	(75.0)
No	314	(25.0)
Treatment in the same city of residence		
Yes	1080	(85.9)
No	177	(14.1)
Area		
Countryside	150	(11.9)
Urban area	1107	(88.1)
Institutionalized patients		
No	1204	(95.8)
Yes	53	(4.2)
Cigarette smoking		
No	1165	(92.7)
Yes	92	(7.3)
Comorbidity		
No	1128	(89.7)
Yes	129	(10.3)
Diabetes	79	(6.3)
Lung mycoses	10	(0.8)
Neoplasia	9	(0.7)
COPD	9	(0.7)
Pneumoconiosis	5	(0.4)
Extrapulmonary TB		
No	1020	(81.1)
Yes	237	(18.9)
Pleural	103	(8.2)
Lymph node	62	(4.9)
Genitourinary	8	(0.6)
Bones	12	(1.0)
Ocular	9	(0.7)
Miliary	16	(1.3)
Meningitis	6	(0.5)
Cutaneous	3	(0.2)
Laryngeal	4	(0.3)
Others	14	(1.1)
Chest X-ray examination		
Suspicious	1093	(87.0)
Normal	121	(9.6)
Other/not done	43	(3.4)
Tuberculin testing		
Negative	176	(14)
Weakly positive	29	(2.3)
Strongly positive	246	(19.6)
Not done	806	(64.1)
Case definition		
New case	1071	(85.2)
Treatment after relapse	69	(5.5)
Treatment after abandonment	106	(8.4)
Transferred-in cost	11	(0.9)

^*∗*^Missing data in 19 patients.

**Table 2:** Univariate and multivariate analyses of risk factors associated with TB treatment abandonment.

Characteristics	Abandonment				Univariate		Multivariate	
	Yes *n* = 84	(%)	No *n* = 1173	(%)	OR (95% CI)	*p*	OR (95% CI)	*p*
Age								
<40 years	52	(62.7)	621	(53.8)	1.0			
>40 years	31	(37.3)	534	(46.2)	0.69 (0.43–1.12)	ns		
Gender								
Female	15	(17.9)	374	(31.9)	1.0			
Male	69	(82.1)	799	(68.1)	2.15 (1.18–3.98)	0.007	2.05 (1.15–3.65)	0.015
HIV coinfection								
No	76	(90.5)	1064	(90.7)	1.0			
Yes	8	(9.5)	109	(9.3)	1.03 (0.45–2.27)	ns		
Alcoholic								
No	68	(81.0)	1054	(89.9)	1.0			
Yes	16	(19.0)	119	(10.1)	2.08 (1.12–3.83)	0.011	—	ns
Literacy								
Yes	62	(73.8)	881	(75.1)	1.0			
No	22	(26.2)	292	(24.9)	1.07 (0.63–1.82)	ns		
Treatment in the same city of residence								
Yes	70	(83.3)	1010	(86.1)	1.0			
No	14	(16.7)	163	(13.9)	1.24 (0.65–2.32)	ns		
Area								
Countryside	8	(9.5)	142	(12.1)	1.0			
Urban area	76	(90.5)	1031	(87.9)	1.31 (0.60–2.99)	ns		
Institutionalized								
No	80	(95.2)	1124	(95.8)	1.0			
Yes	4	(4.8)	49	(4.2)	1.15 (0.34–3.42)	ns		
Extrapulmonary TB								
No	78	(92.9)	942	(80.3)	1.0			
Yes	6	(7.1)	231	(19.7)	0.31 (0.12–0.76)	0.005	0.33 (0.14–0.76)	0.010
Severe extrapulmonary TB^*∗*^								
No	83	(98.8)	1103	(94.0)	1.0			
Yes	1	(1.2)	70	(6.0)	0.19 (0.00–1.12)	ns		
Diabetes								
No	83	(98.8)	1095	(93.4)	1.0			
Yes	1	(1.2)	78	(6.6)	0.17 (0.00–0.99)	0.046	—	ns
Cigarette smoking								
No	76	(90.5)	1089	(92.8)	1.0			
Yes	8	(9.5)	84	(7.2)	1.36 (0.59–3.05)	ns		
Comorbidity								
No	81	(96.4)	1047	(89.3)	1.0			
Yes	3	(3.6)	126	(10.7)	0.31 (0.06–0.96)	0.036	—	ns
Treatment after relapse								
No	81	(96.4)	1107	(94.4)	1.0			
Yes	3	(3.6)	66	(5.6)	0.62 (0.12–1.97)	ns		
Treatment after abandonment								
No	64	(76.2)	1087	(92.7)	1.0			
Yes	20	(23.8)	86	(7.3)	3.95 (2.20–7.05)	<0.001	3.41 (1.96–5.96)	<0.001

^*∗*^Meningitis, miliary, pericarditis, peritonitis, bilateral or extensive pleural effusion, spinal, genitourinary, and intestinal types.

ns: not significant.

**Table 3:** Univariate and multivariate analyses of risk factors associated with death during TB treatment.

Characteristics	Death				Univariate		Multivariate	
	Yes *n* = 146	(%)	No *n* = 1111	(%)	OR (95% CI)	*p*	OR (95% CI)	*p*
Age								
<40 years	48	(34.0)	625	(57.0)	1.0			
>40 years	93	(66.0)	472	(43.0)	2.57 (1.75–3.77)	<0.001	2.61 (1.76–3.85)	<0.001
Gender								
Female	38	(26.0)	351	(31.6)	1.0			
Male	108	(74.0)	760	(68.4)	1.31 (0.87–1.98)	ns		
HIV coinfection								
No	106	(72.6)	1034	(93.1)	1.0			
Yes	40	(27.4)	77	(6.9)	5.07 (3.22–7.97)	<0.001	6.01 (3.78–9.56)	<0.001
Alcoholic								
No	127	(87.0)	995	(89.6)	1.0			
Yes	19	(13.0)	116	(10.4)	1.28 (0.74–2.21)	ns		
Literacy								
Yes	89	(61.0)	854	(76.9)	1.0			
No	57	(39.0)	257	(23.1)	2.13 (1.46–3.10)	<0.001	1.88 (1.27–2.78)	0.002
Treatment in the same city of residence								
Yes	119	(81.5)	961	(86.5)	1.0			
No	27	(18.5)	150	(13.5)	1.45 (0.90–2.33)	ns		
Area								
Countryside	12	(8.2)	138	(12.4)	1.0			
Urban area	134	(91.8)	973	(87.6)	1.58 (0.83–3.09)	ns		
Institutionalized								
No	145	(99.3)	1059	(95.3)	1.0			
Yes	1	(0.7)	52	(4.7)	0.14 (0.00–0.83)	0.024	—	ns
Extrapulmonary TB								
No	116	(79.5)	904	(81.4)	1.0			
Yes	30	(20.5)	207	(18.6)	1.13 (0.72–1.77)	ns		
Severe extrapulmonary TB^*∗*^								
No	130	(89.0)	1056	(95.0)	1.0			
Yes	16	(11.0)	55	(5.0)	2.36 (1.26–4.39)	0.003	2.33 (1.24–4.38)	0.009
Diabetes								
No	136	(93.2)	1042	(93.8)	1.0			
Yes	10	(6.8)	69	(6.2)	1.10 (0.52–2.29)	ns		
Cigarette smoking								
No	139	(95.2)	1026	(92.3)	1.0			
Yes	7	(4.8)	85	(7.7)	0.61 (0.23–1.34)	ns		
Comorbidity								
No	121	(82.9)	1007	(90.6)	1.0			
Yes	25	(17.1)	104	(9.4)	2.00 (1.21–3.29)	0.004	—	ns
Treatment after relapse								
No	131	(89.7)	1057	(95.1)	1.0			
Yes	15	(10.3)	54	(4.9)	2.24 (1.17–4.22)	0.007	1.95 (1.01–3.75)	0.046
Treatment after abandonment								
No	132	(90.4)	1019	(91.7)	1.0			
Yes	14	(9.6)	92	(8.3)	1.17 (0.62–2.19)	ns		

^*∗*^Meningitis, miliary, pericarditis, peritonitis, bilateral or extensive pleural effusion, spinal, genitourinary, and intestinal types.

ns: not significant.
